# Three-Dimensional Reconstruction of National Traditional Sports Cultural Heritage Based on Feature Clustering and Artificial Intelligence

**DOI:** 10.1155/2022/8159045

**Published:** 2022-09-08

**Authors:** Xuefeng Wang, Zhewei Liu

**Affiliations:** Department of Sports, Jiangsu University of Science and Technology, Zhangjiagang 215600, Jiangsu, China

## Abstract

The development of three-dimensional reconstruction technology with cultural heritage in traditional sports allows an accurate portrayal of this aspect of life. Cultural heritage can be documented, recovered, and shown with the tools and techniques. It is an important aspect of self-determination and the ability of marginalized groups to engage in social and cultural life fully. The most challenging aspects of national cultural sports heritage in reconstruction, including armed conflict and war, improper disposal, pollution, poaching, natural disasters, and destruction of heritage sites, have been determined. This article introduces (SCH-AI) new technology for sports cultural heritage (SCH), which develops an improved artificial intelligence (AI) that helps reduce armed conflict and war and promotes tolerance toward religions and people. Athletics improves by bringing individuals together in a social and economic context. Sports engagement boosts community health and productivity. When a clustering algorithm is used, it analyses the data that were provided identifying organic clusters or patterns in the space of features. Clustering is the process of dividing data into distinct subsets. The scenario depicts a strength and conditioning coach who wishes to establish alternative training in sports regimens for players with varying refinement sprint timing. The research of SCH-AI has many advantages; accessing traditional games is regarded as the ideal platform for promoting peace, harmony, and cultural heritage preservation. Sports are also part of the steps to recognize and preserve the rights of people, and it is the protection of their cultural legacy. Cultural heritage gives users a sense of belonging and harmony within a community and a greater understanding of earlier generations and their history.

## 1. Introduction

The world is paying more attention to cultural heritage protection with the advancement of multimedia and graphics image processing technology. The rise of virtual reality technology and the rapid development of cultural heritage protection has taken on a new form [1]. Three-dimensional modelling and printing of museum objects are increasingly important in public engagement and education, perhaps permitting more democratic access to museum collections and introducing new cultural heritage stakeholders [2]. In current times, culture continues to grow, and this article examines the digital protection and inheritance of culture. The study focuses on the features of cultural legacy and the use of a digital database. The forms and methods of cultural heritage distribution are examined to better understand and verify culture is spread in digital form [3]. The evolution of modern digital technology has substantially facilitated the use of digital cameras in various scientific applications, including cultural heritage documentation. Many cultural-historical objects are found at the bottom of bodies of water. Therefore, digital imagery has gone underwater as well [4]. The usage of immersive virtual reality systems in museums is a relatively new trend since new interactive technology has inevitably touched conventional sciences and arts. It is especially true in the case of novel interactive technologies that captivate the general public, such as virtual reality, which has always been the case [5].

Traditional grounding refers to people's cultural legacies, defining human desire, memory confidence, and future trends. The significant contributions to history and the preservation of cultural traditions, along with policies that might help data economic growth in the community, municipal, and global systems may be sustained [6]. The development of cultural heritage and intangible cultural assets will be guided by the integration of digital technology. This study lists and evaluates various intangible cultural heritage and scholarly directions and the current state of intangible cultural heritage in Hainan [7]. The key technology to virtual reality modelling is rebuilding of complicated information using 3D model scenes with a in a digital world; it is not only considered a significant tool for design and planning, construction, and creativity, but it also helped save history and culture and also become a key tool for remodelling and managing historic buildings [8].

Multimodal serious games can increase the visitor's experience by creating an immersive environment. It is considered a way to create smart legacy displays which may be made more interesting by making use of these techniques and merging methods based on delivering a multisensory, virtual, and immersive virtual reality immersion [9]. The development of the implication and denotation of intangible cultural heritage materials, and often as a way to reconstruct the intangible cultural asset classification system, is the goal of animation abstract. The process of producing and realizing nationality intangible cultural heritage resources through animation is described in this research [10]. We demonstrate our work on storytelling-based apps for interactive virtual cultural assets and demonstrate that users value this presentation format, which they see as bringing 3D geometry to life [11].

A 3D reconstruction model of micro-objects is reconstructed with outside dimensions ranging from a few hundred microns to many two-dimensional photographs of an item acquired from various angles. Data acquisition includes a digital microscope that collects still pictures at a resolution of pixels and a computer-controlled turntable that rotates the samples [12]. Incorporating virtual and augmented with AI technology and other methods based on physical items like exhibitions and visual information can help the general public better understand and appreciate the sports cultural heritage. It is now possible to acquire cultural knowledge through serious games, which are video games with educational goals [13]. Several types of cultural heritage may be found in ancient objects. Three-dimensional stereo technology can enhance artifact investigation activities to help the younger generation learn from cultural heritages [14]. Sports educational programming approaches for transmitting cultural heritage and studying the past are emerging at a professional level technology in relation to sports, a part of our history [15]. Upon investigation of the feature clustering algorithm, using this thorough approach, 3D-dimensional reconstruction data may be gathered. The limb movement and expression capture technology achieve the perfect balance and is extensively utilized in animation and film production [16]. Cultural heritage in sports may be studied in three-dimensional reconstructions to develop new technologies for capturing data and the subsequent transformation of these features clustering rich historical material into interactive digital experiences [17]. The nation's sports culture was passed down through the generations. Besides, it was a significant aspect of the Chinese nation's traditional culture, reflecting its unity and national spirit. Concerning Province's nationwide distribution, this study argues that college students should be educated in national sports culture and traditions [18]. Many aspects of a team's effectiveness and efficiency are influenced directly or indirectly by the group's culture. Team members are expected to abide by the culture's established standards of acceptable behaviour, either openly or tacitly [19].

Diversity in sports is critical because it fosters innovation, new ideas, and possibilities for advancement. Good sportsmanship is one of the most prevalent characteristics that emerge in our athletes due to exposure to various cultures [20]. This study focuses on traditional sports and games (TSG) as the cultural component on European character in an historical context. Researchers rely on information from both historical and present studies of TSG in chosen nations to support their findings [21]. Traditional sports are deeply ingrained in the fabric of American life. It is possible to protect and safeguard our principles of equality, if we guarantee maintenance and protection and ensure our subsequent generations have an involvement in them. Moreover, sports of the ancient variety may foster a greater understanding of other cultures. For a clustering characteristic, the statistics for that cluster are summarised. Many relevant statistics may be derived from a cluster using the clustering feature. One may use this method to find fascinating patterns in data, such as groupings of consumers based on their purchasing habits [22].

Quantitative characteristics of the item, such as size or the object's position to other objects in the picture, may only be inferred through reconstruction. It is the purpose of feature clustering to identify groupings or clusters in a dataset that are distinct. Machine language algorithms are used to construct groups of objects with similar qualities, and the tool uses this technique to build groups of items with similar features [23]. A people's values, beliefs, and ambitions are shaped and reflected in their culture and heritage, which helps to define their sense of national identity. The preservation of our cultural legacy is essential to our survival as a people [24]. Sports is far more than just a physical competition, and it is also a way for people to express their cultural and national identities. Attending and supporting various athletic events helps people strengthen their sense of belonging to their communities. In artificial intelligence, sports cultural heritage is used as the mechanisms through which the humanity works. Natural language processing, speech synthesis, and machine vision are examples of intelligent machines with AI applications [25].

The main contribution of the research is as follows:

- A feature clustering tree is an efficient representation of the dataset in which each leaf node corresponds to an individual cluster.
- An artificial intelligence (AI) system is the one that executes living beings on a system or automaton under the guidance of a program.
- Analysing SCH-AI is used in sports to improve performance and health. Athletes can avoid major injuries thanks to devices that gather information about strain and tear levels. It is possible for AI to assist teams in developing their strategies and tactics.
- The goal of SCH-AI for cultural heritage in sports is to give people and organizations working to preserve and enhance cultural heritage the tools they need to do their sports better.

The remainder of the article is as follows: Section 2 indicates a literature review on improving the use of sports cultural heritage SCH-AI.  Section 3 denotes the improvement of 3D reconstruction in national sports with features clustering in AI, Section 4 mentions results and discussion on cultural sports, and Section 5 presents experimental analysis of sports cultural heritage and concludes this work.

## 2. Related Research Work on Sports Cultural Heritage and Its Existing Problems

Gomez et al. initiated that cultural values (CVs) and social practices are intertwined regarding resource protection and exploitation in the oceans, which has led to disputes. An extensive literature analysis, questionnaires [26], and in-depth interviews provide the basis for this study, which highlights the key policy, socioeconomic, environmental, and cultural motivators. According to the findings of this study, promoting social-ecological values, which must be assessed through the prism of environmental ethics, is almost as important as the system of governance.

Dharmadasa and Hp proposed sporting activities are performed in facilities that have been specifically created for this goal. Cricket facilities in Sri Lanka are more numerous than in other countries [27]. Having a surprisingly large number of facilities, the feature clustering algorithm devoted to cricket makes sense, given cricket's prominence as Sri Lanka's most internationally recognized sport. The research looks at methods by that Sri Lankan authorities could improve the cultural heritage (CH) supplied to indigenous and foreign spectators at sporting venues.

He et al. detailed that traditional culture may live in harmony with technological administration in a community. Traditional wisdom (TW) and experience should be incorporated into the current government to improve the quality of life for citizens [28]. As a part of the modernization of government, the preservation of sports cultural heritage SCH-AI is a rich traditional culture that serves as a basis for that process. As a beginning point for governance in society, traditional cultural legacy must be given the meaning of the times by the current administration.

Li et al. introduced the application of information technology (IT) in sports, and they aimed to provide students with a possible new method of college training and educational guidance to improve the practical quality and professional ability of college sports athletes [29]. Semisupervised algorithms for motion input and interactive virtual scenes are presented in this research. Combining artificial intelligence (AI) technology with traditional sports not only enhances its efficacy but also eliminates the time and space restrictions associated with sports training, encouraging students to get a deeper understanding of the issues faced in education and training.

Wang et al. proposed sporting events are an intangible cultural artifact (ICA) with a specific national flavour. Traditional sports and the development of an efficient communication model will assist in capturing with artificial intelligence (AI), the initiative of intercultural communication of traditional sports culture and shape Hebei's image [30]. Traditional sports culture should be promoted by increasing awareness of intercultural communication, selecting intercultural communication material and expanding the channels of intercultural communication throughout particularly to promote intercultural exchange.

Liu et al. detailed that, for the growth of traditional national sports, the safety of the equipment is a fundamental requirement. Based on this, an optical microscope-based flaw detection technique for national traditional sports equipment is presented [31]. The mean filtering technique minimises the noise in the acquired microscopic pictures of traditional national sports equipment. Then, the phase image processing based on the error correction algorithm is carried out.

McLeod et al. initiated the term rent-seeking which refers to the behaviours of individuals or groups that aim to maximize their financial gain at the expense of society [32]. There is a compelling need for scholars to investigate the emergence and persistence of rent-seeking on sports boards because of its essential destructive nature. The purpose of this research is to determine the exact board members of Indian national sports federations using their positions of authority to seek financial gain.

Applying digital display technology to enhance intangible cultural heritage's virtual depiction, this content intends to the importance of artistic analysis, satisfies information age requirements [33], and conveys the concept of cultural property. The author utilizes historical identity of Dai ceramics legacy as an instance to analyze the current materials' projection mechanism, employing innovation of virtual environment to improve a sensation of actuality and interactivity throughout the display.

Wang introduced the development of nationwide sporting events heritage section of the Yunnan-Vietnam Railroad, breaking an idea of a participant conservation of many nationalities and ethnic groups and re-examining opportunities for major sports activity along the railroad from a historical standpoint [34]. It is critical to maintain China's traditional ethnic culture, including minorities' sports cultural legacy, by creating heritage corridors to protect cultural resources and boost regional economic growth.

Guo et al. detailed the organizational integration and advancement of sports and national fitness as intangible cultural heritage. Traditional folk sports may strengthen people's spiritual lives and the national fitness content system [35], according to the findings of this study. Building a Chinese-style national fitness system helps to preserve the cultural legacy of sports and exploring sports techniques that are appropriate for the country's context are all important steps in ensuring the long-term preservation of sports intangible cultural property.

Wang et al. proposed that the degree of martial arts transmission in the feature clustering algorithm is a symbol of the country's standing as a traditional sports venue [36]. Even the status of a martial arts organization indicates the level of transmission of the martial arts. The World Martial Arts Championship, being the world's most prestigious martial arts tournament, serves as a useful benchmark. Feature clustering can separate the host city into two groups, one local and the other international, and then do a cluster analysis to identify two cluster centres. Compare the two and draw a conclusion. The transmission impact is improved when a martial arts tournament is held in a foreign country.

Rose et al. explored the influence of sports team legacy on sponsorships. A survey of the sports, sponsorship, and five empirical research, revealed five aspects of a sports team's heritage [37]. The first study used qualitative research for item production, followed by two quantitative investigations on sports team legacy. According to the results of two further studies, using the history of a sports team to generate good feelings about a team, a sponsor, and the sponsoring company is effective for both fictional and actual teams.

Research on SCH-AI by enhancing this approach: sport has a positive effect on mood and on your ability to focus. It lowers blood pressure and blood tension and also enhances the quality of one's sleep. Maintaining a healthy weight is made easier by doing SCH-AI. It increases your self-esteem and self-assurance, which are connected to leadership qualities. Sport has a positive impact on elderly people's mental health. Some of the challenges students face during sports workouts include clans, sour losers, cost-consciousness, gamesmanship, weather conditions, and work commitments, which might destabilize a game.

## 3. The Application of 3D Reconstruction of National Sports with Artificial Intelligence

It is possible to create three-dimensional models from several photos using 3D reconstruction. 2D photos are created from 3D sceneries in reverse. With reconstruction, scientists may learn about the object's volume and location with other objects in the scene, which are otherwise impossible to discern from a single view. 3D reconstruction has been used in various computer vision applications, including robotics, reverse engineering, augmented reality, and human-computer interaction. Cultural legacy not only promotes long-term a rise in the local economy, such as an increase in visitors' employment, but also strengthens the meaning belonging to self-worth by the people living in the area.

### 3.1. Artificial Intelligence Is Used for Sports Cultural Heritage in Reconstruction

**Figure 1:**
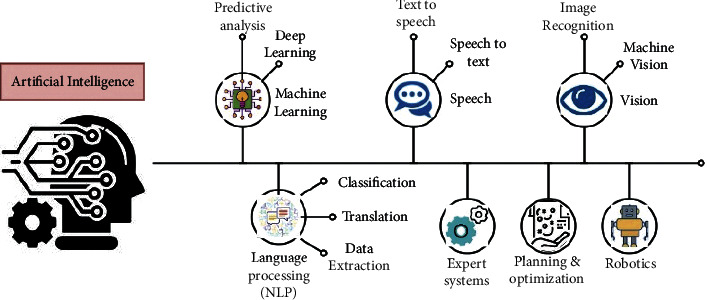
Artificial intelligence in sports cultural heritage.

Figure 1 describes the artificial intelligence in different processes of sports cultural heritage.

Incorporating artificial intelligence (AI), athletics has already started transforming the sport and raising the bar. The introduction of artificial intelligence in sports means that the game's strategy, how it is played, and the audience involved will likely alter in the future. Football, soccer, baseball, basketball, tennis, and other sports are already seeing trends like today. Even in the locker room, artificial intelligence has an influence. Television broadcast highlights and replays more quickly, which gives teams a greater understanding of their opponents. A new road to sports success is being opened up by artificial intelligence as well. By connecting people and groups, sports positively impact social and cultural life. Sports may assist in resolving differences and stimulate discourse, which helps break down injustice, assumptions, cultural differences, ignorance, intolerance, and discrimination. Recovering from injury may be harder for the elderly due to decreased flexibility, strength, and general fitness levels. Golf, bowling, and cycling are popular among the elderly since they are less strenuous and require less physical effort. Sports can make them strong by doing regular activities.

#### 3.1.1. The Derivatives of Artificial Intelligence in Cultural Heritage

(1) $$\begin{array}{c} A\left(X\right)=\frac{xn^{2}+N}{2x_{n}}=\frac{\pi }{2}\left(x_{n}+\frac{N}{x_{n}}\right)+\left(\frac{\left(p-1\right)x_{r}+a}{pxr_{n}}\right)x_{y^{2}}. \end{array}$$

As shown in (1), a form of advanced analytics described *xn*^2^+*N*/2*x*_*n*_ as predictive analytics uses historical data, together, *π*/2(*x*_*n*_+*N*/*x*_*n*_), with statistical modelling, data mining, (*p* − 1)*x*_*r*_+*a*, and machine learning, to forecast the outcomes of future events. In investigating trends in these data, ((*p* − 1)*x*_*r*_+*a*/pxr_*n*_)*x*_*y*^2^_ accompanies the use of sports culture.

(2) $$\begin{array}{c} B\left(X\right)=2sin\frac{ph}{2}*cos\theta \left[px+q+\frac{\left(ph+\pi \right)}{2}\right]\sum x\frac{\delta y}{\delta x}\sqrt{\frac{h}{1+hx+x^{2}}}\;. \end{array}$$

As shown in (2), image recognition-based cos  *θ* photographs is software that can recognize items, places, people, words, and activities. In order to recognize images, computers can use machine vision technology, cameras, and artificial intelligence software.

(3) $$\begin{array}{c} C\left(X\right)=\Delta logf^{x}=log\left(1+\frac{\Delta f\left(x\right)}{f\left(x\right)}\right)+\Delta \left[\frac{f\left(x\right)}{g\left(x\right)}\right]*\int \frac{\Delta f\left(x\right)}{f\left(x\right).f\left(x+1\right)}. \end{array}$$

As shown in (3), computer vision can detect objects, facial emotions, cuisine, natural landscapes, and sports; among other things, for blind users, picture recognition can translate visual information and flag unpleasant or insensitive images in addition to labeling persons in photographs.

### 3.2. Reconstructive Analysis of Culture in Sports

**Figure 2:**
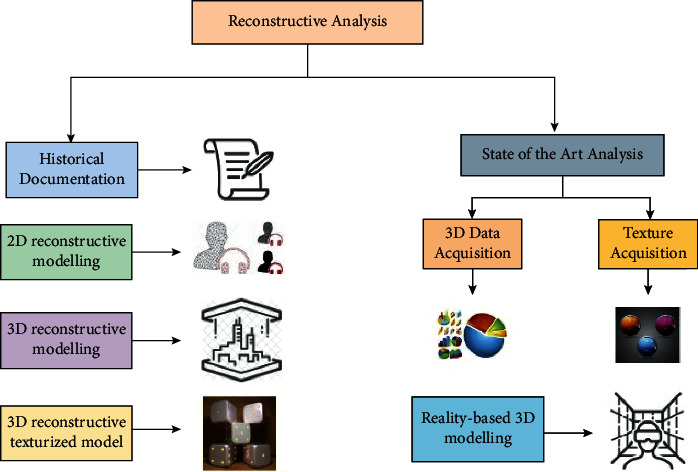
3D reconstructive analysis in sports.

Figure 2 denotes the 3D reconstructive analysis of sports culture in machine learning.

Figure 2 shows that organizations can better use their data by implementing artificial intelligence (AI) in sporting events purposes. Projecting the destiny may assist with everything from the selection of players through the selling of products in a sports business. This sophisticated methodology, based on artificial intelligence, has demonstrated promising outcomes regarding categorization and forecasting tasks. Sports prediction is a growing industry that requires accurate forecasting because of the enormous money involved in betting. More emphasis is being placed on acquiring an advantage over the opponent in every method possible in sports. In the past, this has been accomplished by developing training methods to increase physical performance while decreasing injury risks. A play or game's outcome may be predicted by entering information into a mathematical model, which is exactly what sports analytics is about. Analytical tools are used by front offices to emphasize player development, while coaches utilize them to scout opponents.

#### 3.2.1. The Derivatives of Reconstructive Analysis in Sports

(4) $$\begin{array}{c} D\left(X\right)=log\left(1+\Delta \right)=log2\frac{1}{E}+\sqrt{1+\frac{\delta^{2}}{4}}=\left(\frac{E+E^{-1}}{2}\right)\Delta^{3}Y_{2}\;. \end{array}$$

As shown in (4), sports have a long and illustrious history, dating back more than three thousand years. Historically, a lot of the first forms of competitive sports featured the use of weapons like spears, stakes, and boulders and also one-on-one combat amongst players.

(5) $$\begin{array}{c} E\left(X\right)=y\left(x_{n}+ph\right)=y_{n}+\frac{\nabla y_{n}}{h}.ph+\frac{\nabla^{2}y_{n}}{2.h^{2}}ph.\left(p+1\right)hx^{2}. \end{array}$$

As shown in (5), modeling in two dimensions is a method for creating schematic diagrams and layouts that are only two-dimensional. These papers can represent the layout of a location and where things are located, but they do not provide the depth dimension.

(6) $$\begin{array}{c} F\left(X\right)=tan\theta =\frac{vsin\alpha }{vcos\alpha -u}*\sqrt{\frac{dy}{dx}=\frac{\delta y}{\delta x}}\int \int \frac{\partial y}{\partial x}\;. \end{array}$$

As shown in (6), in general, 2D models are better at depicting flow and velocity distributions. 3D texturing is the process of describing the way light interacts with a 3D object using a 2D picture. Adding texture to a 3D model may be accomplished using a variety of tools and methods included in various software packages. A 3D animation pipeline's texture stage involves unwrapping, painting, and shading textures and rendering.

**Figure 3:**
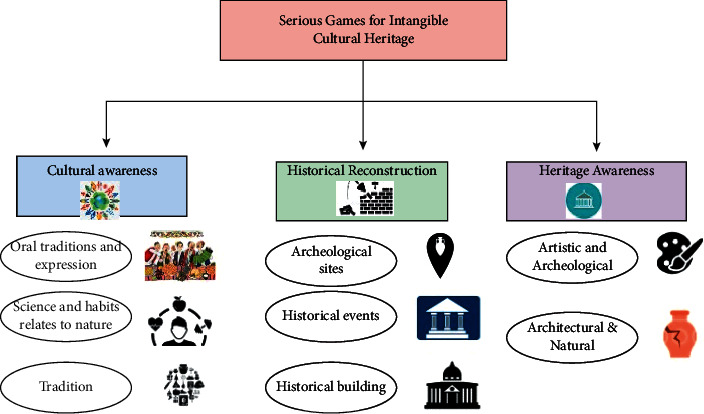
Mathematical framework in intangible cultural heritage.

Figure 3 shows that one of the many benefits of using Intangible cultural heritage is that images on websites may be made more accessible for those with visual impairments, and enormous knowledge graphs can be built to link thousands of concepts together. The use of intangible cultural heritage may substantially improve the efficiency of our workplaces. Freeing up the human labour allows them to concentrate on tasks that need imagination and sensitivity and other abilities, while taking over monotonous or risky duties. There is an increase in self-assuredness due to intangible cultural heritage gains translating into increased resiliency and competitive advantage for organizations that make the required effort up front. As a result, the company will be more equipped to take advantage of possibilities in adjacent industries.

(7) $$\begin{array}{c} Dy_{n}=\frac{xn^{2}+N}{2x_{n}}=\frac{\pi }{2}\left(x_{n}+\frac{N}{x_{n}}\right)f\left(x\right)\;. \end{array}$$

As shown in (7), the values, beliefs, and ambitions of a people are shaped and reflected in their culture and heritage, which helps define their sense of national identity. It must safeguard our cultural legacy if we are to maintain its identity as a nation.

(8) $$\begin{array}{c} G\left(X\right)=\int WsinAcosB+\int \int \left(x+y\right)dx\;=\frac{x^{2}}{2}+x\Delta \frac{dy}{dx}+y. \end{array}$$

As shown in (8), the sport has been credited for fostering deeper social ties and enhancing the self-esteem of the younger generation via participation. Aside from simply entertaining people, sports may also be used to raise awareness about significant social concerns.

### 3.3. Cultural Heritage in Sports with Feature Clustering Algorithm

**Figure 4:**
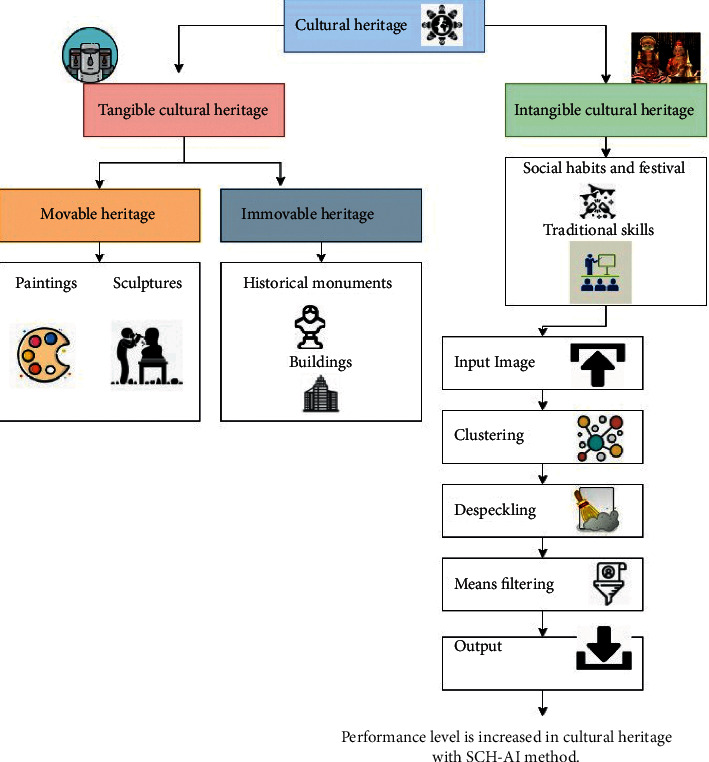
Cultural heritage in sports with feature clustering.

Figure 4 denotes the cultural heritage with a clustering algorithm in sports.

Despeckling is used in terms of developing more effective methods of image recognition providing a sharper representation of a healthcare professional due to widespread usage of equipment that uses sound waves such as ultrasonic screening on the go with portable health apps devices and systems helped by computing. This requires the utilization of effective despeckle filtering.

It is the goal of cultural heritage management to maintain the physical integrity of places while promoting their educational, historical, and cultural aspects simultaneously. Conservation grants, advising and education services, in feature clustering algorithm with cultural heritage identifying and protecting structures and archaeological sites of national significance, and managing historical sites are all part of the mission of the national trust for historic preservation. National heritage location uses a clustering technique based on the data we were dealing with. So, some algorithms must predict the number of clusters in a given dataset, and other algorithms must discover the dataset's shortest possible distance between its observations. Ions are managed to identify, protect, preserve, and convey their worth to future generations. The term tangible cultural legacy describes artifacts that may be kept or handled directly. Traditional attire, tools, structures, artwork, monuments, ways of transportation, and even pottery are of cultural heritage.

#### 3.3.1. The Derivatives for the Sports Heritage

(9) $$\begin{array}{c} H\left(X\right)=\sum f\left(x\right)dx=h\left[1+\frac{E-1}{2}\right]f\left(a\right)+\int \mu \delta *\delta +\sqrt{1+\frac{\delta^{2}}{4}}. \end{array}$$

As shown in (9), the community's intangible cultural legacy includes behaviours, manifestations of knowledge, and abilities recognized as part of its cultural history by the community and also by individuals. The importance of our intangible cultural legacy cannot be overstated because it connects people to our history, present, and future.

(10) $$\begin{array}{c} I\left(X\right)=\mu Y_{X}=\frac{\Delta^{2}}{E}+\left(\frac{\Delta^{2}u_{x}}{Eu_{x}}\right)*\int \emptyset \left(E\right)u_{x}=f\left(x\right)\int \int \frac{1}{\emptyset \left(1+\Delta \right)}f\left(x\right). \end{array}$$

As shown in (10), the value of the collective *μY*_*X*_ intangible cultural legacy cannot be overstated. It fosters a sense of belonging and community among its members and a sense of belonging to society as a whole. Moving and immovable forms of tangible heritage are further tangible and intangible. Archaeological records constitute tangible moveable heritage.

(11) $$\begin{array}{c} J\left(X\right)=Dy_{n}=\frac{1}{h}\left(\nabla +\frac{1}{2}\nabla^{2}+\frac{1}{2}\nabla^{3}\right)y_{n}. \end{array}$$

As shown in (11), heritage artifacts that can be moved, whether natural or artificial, are said to be moveable. Underwater or subsurface treasures are not included in the definition of mobile heritage. As a master of deception, the sculpture speaks to our innate desire for narrative and deception.

(12) $$\begin{array}{c} K\left(X\right)=\frac{dy}{dx}=\frac{1}{h}\left(\nabla y_{n}+\frac{1}{2}\nabla^{2}y_{n}+\frac{1}{2}\nabla^{3}y_{n}\right). \end{array}$$

Sculpture connects ideas, expresses individuality, and fosters social cohesion. The sculpture is a window into the culture at the period it was created.

### 3.4. Mathematical Analysis of Intangible Cultural Heritage Reconstruction with the National Sports

**Figure 5:**
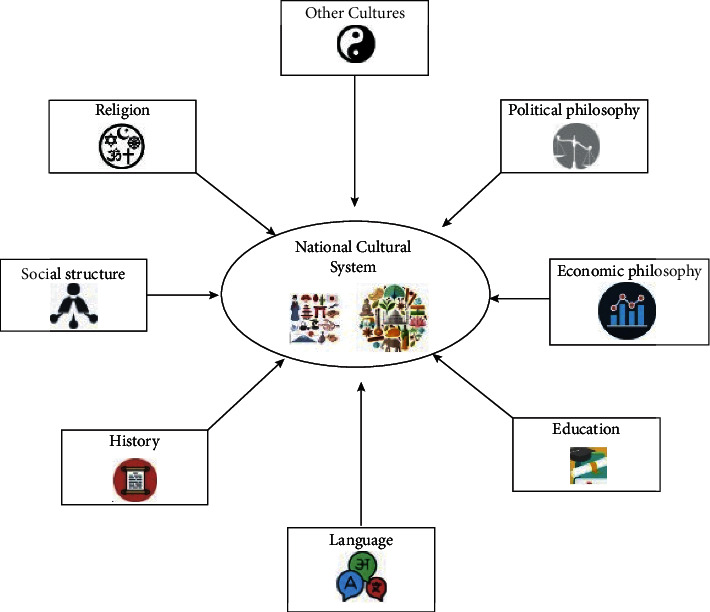
The mathematical analysis of cultural heritage reconstruction with national sports.

Figure 5 shows sports cultural heritage on (SCH-AI) can contribute to a more cohesive society and a more diverse society. Communities and groups are shaped by their social practices, rituals, and celebrations, which may contribute to a more cohesive society. The importance of our sports cultural legacy cannot be overstated because it connects everybody to the history, present, and future. People must understand the relevance of both and conserve all aspects of our cultural heritage. The value of the collective sports cultural legacy cannot be overstated. A sense of belonging to (SCH-AI) a group and society at large is facilitated by this practice. Even in today's globalized world, the importance of both material and sports cultures cannot be overstated; people take our values and culture with us everywhere humans go. People must understand the relevance of both and conserve all aspects of our cultural heritage. This stuff makes the world a fascinating place with variety and beauty. Participating in cultural sports can help you slim down or maintain a healthy weight. Physical and well-being will improve due to participating in a sport. Sports can help anyone overcome sadness and anxiety. In sports, people may test-own their limits and achieve desired ambitions. Bone density is increased by physical activity.

(13) $$\begin{array}{c} L\left(X\right)=y_{k}=c_{1}.\left(\sqrt{2}\right)sin\frac{\pi k}{4}+c_{2}\left(\sqrt{2}\right)cos\frac{\pi k}{4}\int \emptyset \left(E\right)u_{x}\;=f\left(x\right)\int \int \frac{1}{\emptyset \left(1+\Delta \right)}f\left(x\right). \end{array}$$

As shown in (13), we denote the sports cultural heritage of reconstruction in national sports with artificial intelligence. The graph derived from the equation is given in Figure 5.

## 4. Experimentation in the Reconstruction of History of Athletic Practices with Clustering Algorithm

The first step in creating a part of the government's athletic culture is to examine the corridor components, which is an essential precondition for the creation. To guarantee that cultural heritage corridors are built in a way that makes sense from the outset, it is necessary to thoroughly describe and analyze the legacy features that will be included in their creation. To minimize issues caused by poorly specified constituent elements, the project's constituent elements were well-defined, specific regional cultural heritage locations were established, and a flawless historic corridor preservation system was established [38]. For a feature clustering characteristic, the statistics for that cluster are summarised. Sports artifacts, events, and locales have been recognized, revered, and safeguarded in the features clustering algorithm like any other cultural legacy for millennia. The company is a community interest business dedicated to promoting the acquisition, preservation, accessibility, and research of sports artifacts in the United Kingdom and beyond. The following are some of the most typical clustering applications. This number, termed as cluster ID, is assigned to each of the clusters following the clustering process. [39]. All of the work will be done by the Ministry of Arts, Athletics, and Travel. The report will be gathered and analyzed by the culture, sports, and tourism, while field investigation and collection of monuments, historical facts [40], and religious artifacts will be done by the Tourism Bureau. An understanding of the ethnic heritage corridor's values and ideals to better grasp major events, records [41], and people in the ethnic area's regional sports culture is well-known. Wrestling culture, as well as a variety of other sports, is an example of this. The cultural heritage corridor includes, among other things, the art of the campfire and various related practices, and the overall performance in reconstruction of sports cultural heritage applications is explained.

Dataset 1 description: data on sports cultural heritage games is the first dataset we wll be looking at. Using these kinds of datasets can help us understand exactly what a sport's season played out. As a result, many individuals desire to discover which sports are easy and which are difficult that is essential will learn from this set of data. A total of 22 folders featuring the label sports name are included in this set which includes golf, tennis, boxing, chess, cricket, fencing, soccer, gymnastics, ice hockey, and kabaddi are just a few of the many sports represented.

**Figure 6:**
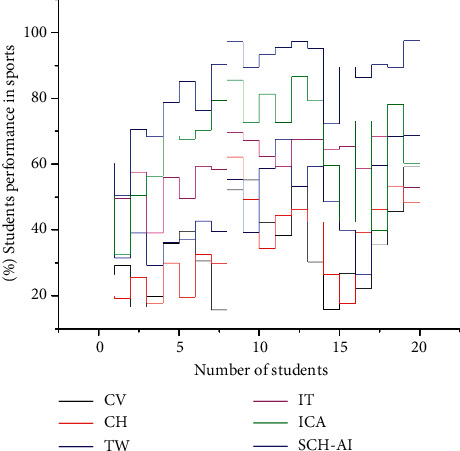
Strategy for the construction of culturally valuable sporting facilities.

Figure 6 shows this article explains the aims of the cultural equity plan, which will be implemented if an organization wants to achieve its goals, which need to lay up a plan that includes everything from resources to policies to outreach to technical help. When towns, cities, counties, regions, and states have a cultural plan, they may better understand their cultural assets, use them to grow their economies, and build stronger communities by concentrating on what makes them great: culture. Cultural heritage management plan resources include tools, forms, and practice notes for creating a plan. Museums and training facilities dedicated to intangible cultural heritage have sprouted up around the country, serving as crucial resources for educating the public about safeguarding this priceless resource.

(14) $$\begin{array}{c} M\left(x\right)=Pcos\theta \;+\mu R\sqrt{\frac{Rsin\alpha -\mu cos\alpha }{cos\theta -\mu sin\theta }}*\sum sin\left(\theta +\beta \right)\;=\frac{tan\alpha sin\beta }{\mu }\int \frac{Psin\left(\alpha +\theta \right)}{cos\left(\alpha -\theta \right)}. \end{array}$$

As shown in (13), sporting events bring people together, fostering a sense of community and enhancing the quality of life for everyone involved. As a result of sports, cultural gaps can be bridged and misunderstandings avoided with the SCH-AI method. Figure 6 explains the value of the derived equation, which results from the value of CV, CH, TW, IT, and ICA in the construction of sports cultural heritage.

Table 1 shows hosting international sporting events, according to some, might be advantageous to governments. Hosting sporting events might attract foreign investment, create jobs, and increase consumer spending. The inherited customs, monuments, artifacts, and culture are part of our heritage. The most important thing is that we use everything humans learned from them in everyday lives. The preservation, excavation, presentation, and restoration of a collection of antiquities are all part of heritage.

(15) $$\begin{array}{c} \;N\left(X\right)=I^{2}m^{2}g^{2}+m^{2}\frac{v^{4}}{r^{2}}\sqrt{\;1+\;\frac{l}{g\delta }}\int 2\pi \left\{\frac{l}{g}\left[1+\frac{1}{4}sin\theta \frac{\gamma \alpha }{2}\right]\right\}\cdot \int \int m\frac{1}{r}*\frac{d}{dt}. \end{array}$$

As shown in equation (15), AI for cultural heritage uses the potential of AI to enable people and organizations devoted by protecting on enrichment of the cultural legacy of sports SCH-AI. Table 1 explains the value of the derived equation, which results from the value of CV, CH, TW, IT, and ICA in the construction of sports cultural heritage.

**Figure 7:**
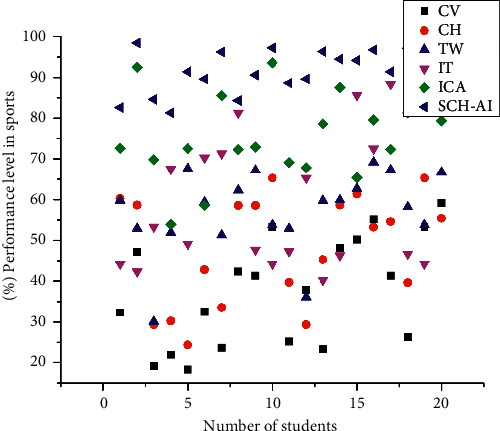
The relevance of national sporting traditions heritage.

Figure 7 shows that sports brings people together in a way that improves the quality of their lives both socially and culturally. Through its ability to foster understanding and debate, sports may help eliminate prejudice, stereotypes, cultural differences, and other forms of harassment. These customs serve as rallying points for supporters and enhance the atmosphere on the field. It is exactly so many sports teams and tournaments have a long history of preserving their traditions. It adds a new dimension to the sport for spectators and participants alike. It is common for customs and trends to serve as a strategy to develop a cultural identity comparable to one's surroundings.

(16) $$\begin{array}{c} O\left(X\right)=\frac{\mathrm{WR}\;\cos A}{2R\;\sin B\;\cos C}+\sum \frac{2\;W}{4\;\sin\theta }*\int W\;\sin A\;\cos B\cdot \int \int \frac{4\;\cos\theta }{2}+\frac{\mathrm{Wb}}{4\mathrm{C\theta}}=\sqrt{\left(\frac{\sin\left(\beta +\alpha \right)}{T}\right)}. \end{array}$$

As shown in (16), in cultural heritage, proper changes have been made throughout time to preserve its historical significance. Many cultures consider games and sports as forms of enjoyment and native culture. Figure 7 explains the value of the derived equation with a number of events and national sports traditions, which results from the value of CV, CH, TW, IT, and ICA in the construction of traditions and customs.

Table 2 shows that constructing a model helps us get insight into the object's qualitative qualities, such as volume and location to other objects. 3D reconstruction in computer vision and computer graphics captures the shape and look of real things. Passive or active approaches can be used to carry out this procedure. Nonrigid or reconstruction refers to varying the model's form to time. Animation, human-computer interfaces (HCI), robotics, source code, augmented reality (AR), and entertainment all fall under the automation category and are just a few areas where rapid and precise 3D reconstruction has been found useful. One of the many types of nursing assessments that community nurses must perform is examining one's family history. Community nurses can acquire essential information about a patient's culture and beliefs through a heritage assessment.

(17) $$\begin{array}{c} P\left(X\right)=\cos\mathrm{\varphi *}\frac{p\;\sin\;\propto }{p+p\;\cos\;\propto }+\sqrt{\frac{\pi }{2}p^{2}}+2p^{2}\cos\alpha +p^{2}-\left(\frac{Q+R}{2P}\right)=\frac{2\;\sin\;\propto \;\cos\beta }{\tan\varphi }. \end{array}$$

As shown in (17), cultural heritage sports with AI signifies maintaining the treasures and memories of a group despite the efforts of outside influences to alter or erase them. Table 2 explains the value of the derived equation with tradition, which results from the value of CV, CH, TW, IT, and ICA in the construction of sports cultural heritage.

**Figure 8:**
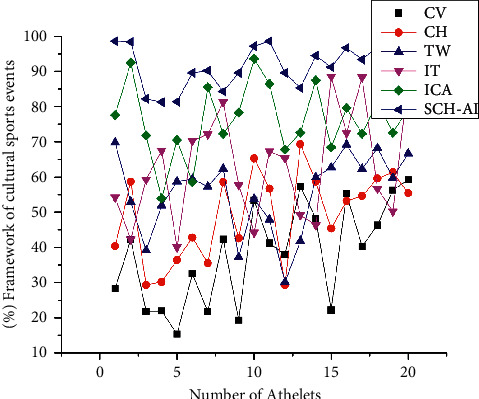
Comprehensive framework of sports with artificial intelligence.

Figure 8 shows that the player technique can be improved using sensor technologies and artificial intelligence (AI). An increasing number of athletes are benefiting from using artificial intelligence in the form of real-time feedback and individualized training plans. The use of artificial intelligence in sports with (SCH-AI) has the potential to improve health and fitness. Athletes can be protected from injury using wearable applications to track their wear and strain. Before and during the game, artificial intelligence has a significant influence on strategy. Computer analysis is increasingly a factor in making decisions regarding the starting line-up before and during a game. Even the location and motion of players, as well as the spin, speed, and placement of serves, may be used to improve sports performance using artificial intelligence.

(18) $$\begin{array}{c} Q\left(X\right)=\text{tan D}\left(\frac{\pi }{4}-\frac{\alpha }{2}\right)+\left(\frac{1+\mu }{2}\right)*\sqrt{\frac{\cos\;\theta }{\mu }-1\;\sin\frac{\pi }{2}}\cdot \int \left[\frac{W}{2a}\left\{d+x-\frac{\tan\;\sigma }{2a+x}\right\}\right]-\int \int \frac{u\;\sin\;\alpha }{u+u\;\cos\;\theta }. \end{array}$$

As shown in equation (18), AI is used in sports to boost performance and health, athletes can avoid serious injuries by using (SCH-AI) technology, and it helps teams shape strategies, tactics, and maximize their strengths. Figure 8 explains the value of the derived equation with tradition, which results from the value of CV, CH, TW, IT, and ICA in the construction of sports cultural heritage.

## 5. Conclusion

This paper discussed the three-dimensional reconstruction of national traditional sports cultural heritage, which could be enhanced by applying the SCH-AI-assisted clustering algorithm. Cultural protection of national history serves to safeguard the country's past, reputation, and source of satisfaction. SCH-AI has basic-findings that reduce armed conflict and war, improper disposal, pollution, poaching, and natural disasters. This article focuses on a new technological instructional application that combines science, history, and archaeology. By using gaming platform tools and participating in constructing the virtual environment, students at universities utilize e-learning to improve their education by actively participating in practically every implementation stage and analyzing the degree of involvement. The importance of SCH-AI in sports cultural heritage on 3D reconstruction can be preserved and successfully applied to AI. As a result, greater attention should be paid to the potential applications of AI outside of the realm of technology. Artificial Intelligence AI is a new and quickly expanding technology, and it will have an impact on more individuals as time goes on. In future, quantization techniques will be used in the future to improve the model size for portability to edge devices. It is anticipated that the IHDS dataset will be improved and used for the 3D reconstruction of heritage sites to create walkthroughs, enabling the project to achieve a second goal.

## Figures and Tables

**Table 1:** The construction of sporting events in the clustering algorithm.

Value of sports cultural heritage	CV	CH	TW	IT	ICA	SCH-AI
1	28.3	40.3	69.8	54.2	77.6	98.6
2	42.2	58.7	52.9	42.3	92.5	98.4
3	21.8	29.3	39.1	59.3	71.8	82.2
4	21.9	30.2	51.9	67.5	53.9	81.3
5	15.3	36.4	58.7	40.1	70.5	81.4
6	32.5	42.8	59.4	70.3	58.6	89.6
7	21.7	35.5	57.3	72.1	85.5	90.2
8	42.3	58.6	62.3	81.3	72.3	84.3
9	19.3	42.6	37.2	57.7	78.3	89.6
10	53.3	65.3	53.8	44.2	93.6	97.2
11	41.2	56.7	47.9	67.3	86.5	98.6
12	37.9	29.3	30.1	65.3	67.8	89.6
13	57.3	69.3	41.8	49.2	72.6	85.3
14	48.2	58.7	59.9	46.3	87.5	94.5
15	22.2	45.4	62.7	88.6	68.4	91.2
16	55.2	53.2	69.2	72.5	79.6	96.7
17	40.3	54.6	62.3	88.3	72.3	93.4
18	46.3	59.6	68.2	56.7	81.3	97.1
19	56.3	61.3	59.8	50.2	72.6	96.3
20	59.2	55.4	66.7	87.6	79.4	94.2

**Table 2:** 3D reconstructions in the protection of cultural heritage in a systematic study.

Value of reconstruction	CV	CH	TW	IT	ICA	SCH-AI
1	32.3	60.3	59.8	44.2	72.6	82.6
2	47.2	58.7	52.9	42.3	92.5	98.4
3	19.2	29.3	30.1	53.3	69.8	84.6
4	21.9	30.2	51.9	67.5	53.9	81.3
5	18.3	24.4	67.7	49.1	72.5	91.4
6	32.5	42.8	59.4	70.3	58.6	89.6
7	23.7	33.5	51.3	71.3	85.5	96.2
8	42.3	58.6	62.3	81.3	72.3	84.3
9	41.3	58.6	67.2	47.7	72.9	90.6
10	53.3	65.3	53.8	44.2	93.6	97.2
11	25.2	39.7	52.9	47.3	69.1	88.6
12	37.9	29.3	36.1	65.3	67.8	89.6
13	23.3	45.3	59.8	40.2	78.6	96.3
14	48.2	58.7	59.9	46.3	87.5	94.5
15	50.2	61.4	62.7	85.6	65.4	94.2
16	55.2	53.2	69.2	72.5	79.6	96.7
17	41.3	54.6	67.3	88.3	72.3	91.4
18	26.3	39.6	58.2	46.7	81.3	97.1
19	53.3	65.3	53.8	44.2	82.6	96.3
20	59.2	55.4	66.7	87.6	79.4	94.2

## Data Availability

The data that support the findings of this study are available from the corresponding author upon reasonable request.
